# Mitochondrial DNA release and inflammation in mitochondrial disease pathogenesis

**DOI:** 10.1093/brain/awag037

**Published:** 2026-02-02

**Authors:** Marton Szabo, Daniel Lagos, Emily Cross, Jack J Collier, Rita Horvath

**Affiliations:** Department of Clinical Neurosciences, University of Cambridge, Cambridge CP2 0PY, UK; Department of Clinical Neurosciences, University of Cambridge, Cambridge CP2 0PY, UK; Department of Clinical Neurosciences, University of Cambridge, Cambridge CP2 0PY, UK; Department of Clinical Neurosciences, University of Cambridge, Cambridge CP2 0PY, UK; Department of Clinical Neurosciences, University of Cambridge, Cambridge CP2 0PY, UK

**Keywords:** mitochondrial DNA, mitochondrial DNA release, primary mitochondrial diseases, sterile inflammation, mitochondria derived vesicle, pathogen-associated molecular patterns

## Abstract

Primary mitochondrial diseases (PMDs) affect ∼1 in 4300 individuals, yet mitochondrial dysfunction is also a hallmark of common inherited and acquired disorders. Although advances in genomics now allow molecular diagnosis in the majority of mitochondrial diseases, treatment remains largely supportive, leading to progressive disability and early mortality. Despite progress in gene-modifying approaches, no approved therapies exist for the majority of mitochondrial diseases, and none of the recent trials has met its primary end point, underlining the urgent need for innovative therapeutic strategies.

Patients with PMDs have highly variable phenotypes, further complicated by increased susceptibility to infections, chronic inflammation and metabolic abnormalities. Recently, it has become evident that certain mitochondrial pathologies, including the loss of mitochondrial membrane integrity, impaired mitochondrial DNA (mtDNA) maintenance, quality control defects or respiratory chain defects, result in the release of mtDNA into the cytosol. Infections or metabolic changes also trigger the release of mtDNA, leading to the activation of a sterile innate immune response and interferon signalling. Free mtDNA acts as a pathogen-associated molecular pattern (PAMP), activating innate immune pathways such as the cGAS–STING axis, initiating a sterile inflammatory response. This can be followed by the extracellular release of mtDNA to convey the inflammatory response systemically to communicate between cells or across organs. However, it is unclear whether these pathways worsen the disease phenotype (hyperinflammatory reaction) or, in contrast, rescue the symptoms owing to upregulation of compensatory pathways.

In this review, we summarize recent advances in understanding the mechanism of mtDNA release and how it activates innate immune signalling in PMDs. We also discuss the implications for pathogenesis, clinical phenotypes and therapeutic development. Defining the role of circulating mitochondrial material as a biomarker or therapeutic target is a crucial step for precision medicine approaches in PMDs. These pathways might also have wider implications for common metabolic, inflammatory and neurodegenerative disorders with mitochondrial dysfunction.

## Teaser

Szabo *et al.* review how mitochondrial damage can trigger the release of mitochondrial DNA, activating innate immune signalling pathways. Understanding this process could reveal new disease markers and treatments for mitochondrial diseases as well as for common metabolic and neurodegenerative disorders.

## Mitochondrial DNA release: a new signalling mechanism in mitochondrial diseases

Mitochondria are highly specialized, dynamic organelles that control the cellular bioenergetic state. They possess their own genome, termed mitochondrial DNA (mtDNA), with hundreds to thousands of copies distributed across the mitochondrial network within individual cells. mtDNA encodes 13 essential proteins of the electron transport chain (OXPHOS), two ribosomal RNAs (rRNAs) and 22 transfer RNAs (tRNAs) necessary for intramitochondrial protein translation, with >1200 nuclear-encoded genes providing most of the mitochondrial proteome. Pathogenic variants in mtDNA or nuclear-encoded mitochondrial proteins can lead to the development of primary mitochondrial diseases (PMDs), a diverse group of genetic disorders affecting 1 in 4300 people.^1^ Around 400 genes have been associated with PMDs, which include structural subunits and assembly factors of OXPHOS, mtDNA maintenance proteins, mitochondrial translation, fusion/fission, metabolic pathways and transport processes. PMDs can manifest at any age and usually lead to progressive multisystemic syndromes, affecting the skeletal muscle, brain and heart. Maternally inherited or *de novo* mtDNA mutations may be present in only a fraction of the mtDNA copies in the cell (heteroplasmic mutations) or in all mtDNA copies (homoplasmic mutations). Postmitotic cells and tissues tend to accumulate a high mutation load, leading to clinical manifestations. However, mtDNA heteroplasmy does not fully explain the variable phenotype in PMDs, which can be influenced by additional cell type-specific mechanisms.^2^ Likewise, common nuclear variants (e.g. *POLG*) can result in highly variable disease onset, progression and severity, highlighting the relevance of other contributing factors in the pathophysiology.^3^

Mitochondria are compartmentalized organelles, separated from the cytosol by the outer and inner mitochondrial membranes (OMM and IMM). Severe mitochondrial damage compromising the integrity of these membranes can lead to the release of inner mitochondrial contents into the cytosol via the insertion of pores. A well-known example is the release of cytochrome *c* through Bak/Bax outer membrane pores, which activates the apoptotic cascade. In fact, this discovery transformed our understanding of mitochondria, revealing them to be critical signalling organelles that orchestrate cell fate. Furthermore, it has recently been demonstrated that mitochondrial components, such as mtDNA and mitochondrial RNA (mtRNA), can also be released into the cytosol and act as damage-associated molecular patterns, initiating potent inflammatory responses. Released mtDNA is sensed by the cytosolic dsDNA sensor cGAS, which generates 2′,3′-cGAMP to activate STING and downstream type I interferon responses. Additionally, mtDNA can activate the AIM2 or NLRP3 inflammasome and the TLR9–NF-κB signalling axis, further amplifying inflammation. These responses can trigger cytokine production and pyroptosis, an inflammatory form of programmed cell death. Such phenomena have been reviewed extensively by others, and multiple mtDNA release pathways have been discovered and reviewed extensively elsewhere.^4-6^ This mitochondria-driven inflammatory signalling is increasingly recognized in various conditions with known mitochondrial involvement, including senescence and cardiovascular, autoimmune and neurodegenerative disorders, strengthening its possible role in mitochondrial diseases.

Mitochondrial diseases encompass a diverse and complex spectrum of clinical manifestations. Among the most frequently observed phenotypes are diabetes mellitus, myopathy, cardiomyopathy, dementia, encephalopathy and seizures. Notably, for several of these conditions, inflammation has been identified as a contributing factor in their pathophysiology.^7^ The involvement of inflammation is becoming increasingly evident in PMD, suggesting that immune pathway activation might play a broader role in mitochondrial disease than previously recognized. Indeed, elevated levels of circulating mtDNA have been reported in patients with mitochondrial diseases, along with signs of activation in key inflammatory pathways.^8,9^ Additionally, studies in healthy individuals have demonstrated that mtDNA content within circulating extracellular vesicles is correlated with levels of pro-inflammatory cytokines, reinforcing the link between mitochondria-derived signals and systemic inflammation.^10^ Furthermore, components of the innate immune system, particularly microglia/macrophages and IFN-γ, rather than adaptive immunity, have been recognized as key contributors to symptom development in the *Ndufs4*^−/−^ mouse model of Leigh syndrome.^11,12^ Together, these findings underscore the potential role of innate immune responses in establishing and modulating mitochondrial disease phenotypes and open avenues for further exploration of inflammation-targeted therapeutic strategies.

Here, we aimed to compile the evidence linking the mtDNA release from mitochondria and the consequent activation of sterile cellular innate immune responses in PMDs. We selected a total of 36 publications, 24 of which focused on PMD-related genes and 12 that addressed genes not directly related to PMDs, but still relevant for mitochondrial biology. Selected articles were sorted into broad functional modules of mitochondrial biology (Tables 1 and 2). Our study demonstrates the presence of mtDNA release in several PMDs, underscoring its importance in the pathophysiology and, possibly, in the progression of the disease.^9,13^ A better understanding of these pathways will highlight new targets and potential treatments for these so far incurable and devastating conditions.

**Table 1:** Summary of study characteristics and key findings on sterile inflammation caused by mitochondrial DNA release in PMDs

Reference	Model	Key findings
**Mitochondrial membrane integrity**		
Lepelley *et al.*^14^	ATAD3A patients, *ATAD3a*-KD THP-1 cells	Increased ISG and TNF expression in blood. mtDNA release, type 1 IFN signalling, STING activation via VDAC pores.
**Mitochondrial quality control**		
Sprenger *et al.*^15^	*YME1L* KO mouse retina *YmeIl*^−/−^ MEFs	mtDNA release and cGAS/STING/TBK1 activation. Impaired pyrimidine synthesis drives mtDNA release and immune sensing.
Rodríguez-Nuevo *et al.*^16^	*Opa1* ^−/−^ mouse skeletal muscle	Mitochondrial dysfunction, FGF21 release, NF-κB, pro-inflammatory response. Muscle inflammation at early stages, before macrophage infiltration. Blockage of TLR9 prevented inflammation via NF-κB activation.
Todkar *et al.*^17^	*Opa1*-KO MEFs	Cells selectively prevent the packaging of oxidized mito-protein into EVs. Opa1+SNX9-dependent MDVs are packaged into EVs.
Irazoki *et al.*^18^	*Mfn1* ^−/−^ mice *Mfn1*, *Mfn2*, *Drp1* and *Fis1* KD myoblasts	Mitochondrial fragmentation and activation of the cGAS–STING pathway. Drp1 and Fis1-KO, elongated mitochondria and activation of TLR9-NF-κB. mtDNA in Mfn1-KD cell lines co-localized with early endosome marker Rab5C.
Filograna *et al.*^19^	Dopaminergic neurons in *Mfn2*^loxP/loxP^ mice	Fragmented mitochondria, impaired transport, increased NF-κB-regulated genes. Pro-inflammatory NLPR3 inflammasome expression in glial cells.
Park *et al.*^20^	*Drp1*-KD bone marrow-derived macrophages	Elongated mitochondrial morphology, increased apoptotic and caspase activity. Increased flux of mtDNA to the cytosol, activation of NLRP3 inflammasomes.
Torres-Odio *et al.*^21^	*Clpp*-KO mice and MEFs	Enlarged and aggregated nucleoids, increased mtDNA and TFAM expression. Increased mtDNA release mediated by VDAC pores. Induced cGAS–STING pathway, resulting in type I IFN signalling.
**Mitochondrial DNA maintenance**		
Zhong *et al.*^22^	*Tfam* ^delMye^ mice *Trif*^−/−^ *Myd88*^−/−^ mice	TLRs engage MyD88 and TRIF to trigger CMPK2 transcription. Oxidized cytosolic mtDNA associates with NLRP3 for inflammatory activation.
Chung *et al.*^23^	Tubule-specific *Tfam*-KO mice	Impaired mtDNA packaging, mtDNA release and cGAS–STING activation. Inhibition of STING via KO alleviated kidney fibrosis.
West *et al.*^8^	*Tfam* ^+/−^ MEFs and BMDMs	mtDNA release, cGAS–STING–IRF3 signalling, type 1 IFN and viral resistance. Herpes virus induces mtDNA stress to trigger the antiviral response.
Newman *et al.*^24^	*Tfam* ^+/−^ MEFs and *Bak*^−/−^ *Bax*^−/−^ MEFs	mtDNA replication stress, enlarged TFAM-bound mtDNA release. mtDNA nucleoids before activation of the checkpoint observed in stress. Endosomal rupture during the recycling pathway activates cGAS–STING.
Oka *et al.*^25^	*DNase2a* ^−/−^ mice and mouse cardiomyocytes	mtDNA escapes autophagy, triggers TLR9 sensing and inflammatory response. Inhibiting TLR9 rescues cardiomyopathy and inflammation.
Saito *et al.*^26^	*Mcl-I*/*Dnase2a-* KO mice	Cytosolic mtDNA, interferon production and apoptosis dependent on TLR9. Fatty livers with increased mtDNA release and IFN response.
Jou *et al.*^27^	*TK2*-deficient patients	COX-negative and ragged-red fibres, increased SDH, endomysial inflammation. Lamellar cristae, electron-dense granules and intramitochondrial vacuoles.
Dragoni *et al.*^28^	*RNaseH2A*/*B* mutant primary human lymphoblastoid cells	Altered membrane/cristae organization, VDAC oligomerization, increased ROS. Cytoplasmic mtDNA release.
Milenkovic *et al.*^29^	*Mgme1* ^−/−^ mice	Linear mtDNA fragments. Inflammatory disease symptoms, nephrotic syndrome, weight loss and retinopathy.
Cobo *et al.*^30^	*DNMT3A*/*TET2* mutant human and mouse BMDM	Defective mtDNA integrity owing to decreased TFAM expression. TFAM decrease triggers mtDNA release into the cytosol, and mtDNA is sensed by cGAS/STING and triggers type I interferon response.
Lei *et al.*^31^	*Polg* ^D257A/D257A^ mice	cGAS–STING–IFN1 activation, low NRF2, increased oxidative stress. Blocking IFN1 restores NRF2, oxidative stress, and lowers aerobic glycolysis.
**Mitochondrial transcription and translation**		
Zhao *et al.*^32^	MELAS and CPEO patients	Increased circulating cell-free mtDNA in blood, increased levels of cGAS–STING proteins and enhanced expression of multiple inflammatory cytokines.
**OXPHOS and energy metabolism**		
Baek *et al.*^33^	*Cox10* ^loxP/loxP^ mice *COX10* KO tubular epithelial cells	Increased cytosolic mtDNA content and increased cGAS–STING, enhanced interferon expression, upregulation of pro-apoptotic genes. Mutant mice died prematurely of kidney failure, attributable to depleted glomerular and tubular epithelial function, and autoimmune inflammation.
Cotticelli *et al.*^34^	*FXN-*KD human cardiomyocytes	mtDNA depletion, increased cytosolic mtDNA, mitochondrial dysfunction. cGAS–STING signalling, type I interferon-mediated inflammation.
Aguilar *et al.*^35^	*Ndufs4^(−/−)^* mice	Microglia are key mediators of neuroinflammation. Interleukin-6 does not play a crucial role.
Hanaford *et al.*^36^	*Ndufs4^(−/−)^* mice	Depletion of microglia does not drastically alter disease progression. Evidence that peripheral macrophages are responsible for the CNS phenotype.

+/− = heterozygous genotype; −/− = homozygous knockout; BMDMs = bone marrow-derived macrophages; cGAS = cyclic GMP–AMP synthase; CPEO = chronic progressive external ophthalmoplegia; del = deletion allele; EVs = extracellular vesicles; ISG = interferon-stimulated genes; KD = knockdown; KO = knockout; loxP/loxP = conditional floxed allele used for Cre-mediated recombination; MDVs = mitochondria-derived vesicles; MEFs = mouse embryonic fibroblasts; MELAS = mitochondrial encephalomyopathy, lactic acidosis, and stroke-like episodes; OXPHOS = oxidative phosphorylation; PMDs = primary mitochondrial diseases; ROS = reactive oxygen species; STING = stimulator of interferon genes.

**Table 2:** Summary of study characteristics and key findings on sterile inflammation caused by mitochondrial DNA release in non-PMD models and mutations of unconfirmed significance

Reference	Model	Key findings
**Mitochondrial membrane integrity**		
Chen *et al.*^37^	*SAM50*-KO hepatocytes	Axis formed between SAM50–MICOS–ATAD3a–mtDNA; SAM50 interacts with MIC16 and cardiolipin to maintain membrane integrity. *SAM50* depletion causes cardiolipin leak, BAK/BAX formation, mtDNA release. Paracetamol caused *SAM50* reduction, mtDNA release and cGAS activation.
Liu *et al.*^38^	*Phb1* ^−/−^ mice and *Phb1*-KD macrophages	Inflammation, membrane integrity loss, increased cytoplasmic mtDNA. Cytosolic mtDNA triggers NLRP3 and AIM2 inflammasome. SPG7 and AFG3L2 interaction, mPTP and mtDNA release and inflammation.
He *et al.*^39^	HeLa KO screen	Mitochondrial cristae regulators involved in IFN response (including MICOS, ATP5, SAM and ATAD3A) and phospholipids (cardiolipin and phosphatidylethanolamine). Inflammation mediated by STING is caused by mtDNA release via VDAC pores.
Field *et al.*^40^	FABP5 inhibited regulatory T cells	mtDNA release and IL-10 activate regulatory T cells involved in the tumour microenvironment. Mitochondria change in morphology, cristae architecture and lipid metabolism.
**Mitochondrial quality control**		
Rai *et al.*^41^	*Irgm1* ^−/−^ mice, *Irgm1*^−/−^ MEFs, Macrophages	Autoimmune-like pathology, while MEFs showed increased nucleoids and activation of the cGAS–STING pathway, playing a crucial role in the symptoms. *Irgm1*-deficient macrophages showed TLR7-dependent IFN-1 inflammation.
Irazoki *et al.*^42^	*BNIP3*-KD myoblasts	Increased expression of late endosomal/lysosomal markers Rab7 and LAMP1, but not of early endosomal markers Rab5 and EEA1. Mitochondria in BNIP3 KD co-localize with Rab7 late endosomal marker. Mutant myotubes exhibit an activated NLRP3 inflammasome and TLR9–NFB inflammatory pathway, coupled with co-localization of TLR9 with mtDNA.
Bueno *et al.*^43^	*Pink^−/−^* mice and mouse lung epithelial cells	Tunicamycin-induced endoplasmic reticulum stress facilitated mtDNA release and coupled pro-inflammatory and profibrotic response through the TLR9–NF-κB pathway. Released mtDNA, increased mutation rate driving the inflammatory response.
Yang *et al.*^44^	*Ngly1* ^+/−^ mice and *Ngly1*-KO MEFs and THP-1 cells	Tubular and fragmented morphology with impaired mitophagy. Released mtDNA, activation of inflammatory type I interferon signalling through cGAS–STING and MDA5–MAVS pathways. Nfr1 has an NGLY1-dependent role in mitophagy and mitochondrial quality control.
**Mitochondrial DNA maintenance**		
Al Khatib *et al.*^45^	*TOP1MT* KO MEFs	Fused mitochondrial morphology, increased efflux of mtDNA to cytosol and cGAS–STING activation.
Xian *et al.*^46^	*Ogg1* ^−/−^ mice	mtDNA escapes mitochondria via VDAC and mtPTP, activating cGAS–STING. Oxidized mtDNA is fragmented by FEN1, transported to the cytosol, activating NLRP3.
Kim *et al.*^47^	*EndoG* ^−/−^, *Vdac1*^−/−^, *Vdac3*^−/−^ and lupus mice (Mpj-Fas^lpr^)	Mito with stress releases short mtDNA fragments via VDAC pores in OMM. VDAC N-terminal interacts with mtDNA for oligomerization. VBIT-4 (VDAC oligomerization inhibitor) decreases mtDNA release and IFN signalling.

+/− = heterozygous genotype; −/− = homozygous knockout; cGAS = cyclic GMP–AMP synthase; del = deletion allele; IMM = inner mitochondrial membrane; KD = knockdown; KO = knockout; loxP/loxP = conditional floxed allele used for Cre-mediated recombination; MEFs = mouse embryonic fibroblasts; OMM = outer mitochondrial membrane; PMD = primary mitochondrial disease; STING = stimulator of interferon genes; VDAC = non-selective voltage-gated ion channel VDAC1.

## Mitochondrial membrane maintenance and organization

Mitochondrial membranes define distinct mitochondrial compartments and separate mitochondrial content from the cytosol. Thus, mitochondrial membranes protect mtDNA from cytosolic DNA sensors. Beyond compartmentalization, the OMM acts as a signalling platform that can directly contact other cellular compartments, whereas the IMM invaginates into cristae, which support efficient ATP production, mtDNA maintenance and protein translation.^48^ Both membranes also play important roles in regulating mitochondrial dynamics, turnover and lipid metabolism. It is therefore essential that membrane organization and composition are maintained.

Disruption of the mitochondrial membranes is increasingly recognized as a key driver of mitochondrial content release and the subsequent activation of inflammatory pathways. Several genes that maintain the architecture of both the outer and inner mitochondrial membranes have been implicated in this process, which often involves mtDNA release. These include scaffolding and chaperone proteins that control cristae and mtDNA organization, including the SAM50–MICOS–ATAD3 axis^49,50^ and prohibitin 1 (PHB1),^38^ and lipid metabolism, such as FABP5.^40^ Cellular depletion of SAM50, PHB1 or FABP5 facilitates mtDNA release through the formation of BAK/BAX pores, opening of the mitochondrial permeability transition pore (mPTP), disruption of cardiolipin synthesis (a key lipid of the inner mitochondrial membrane with roles in membrane fusion and stability), and structural disorganization of cristae.^37,39,46,51^ Furthermore, an ATAD3-knockdown line demonstrated mtDNA release to occur via pores formed through VDAC oligomerization, a well-described release mechanism promoting inflammation.^14,47^ Collectively, these alterations trigger inflammation by activating the cGAS–STING pathway. Although, except for *ATAD3A*, these genes have not yet been implicated in PMDs, they underscore the critical importance of mitochondrial membrane integrity in restricting mtDNA release and consequent inflammation, providing important insights for PMDs caused by impaired membrane integrity and lipid metabolism.

Supporting these conceptual advances, a recent CRISPR screen mapping mitochondrial components whose depletion induced cGAS/STING-dependent type I interferon (type I IFN) signalling identified dozens of genes encoding proteins that maintain cristae organization.^39^ Mechanistically, the screen revealed that membrane disruption impaired cristae architecture, triggering VDAC oligomerization, through which mtDNA is released. Some highlighted genes included regulators of cardiolipin metabolism (*TAMM41*), F_1_F_0_-ATP synthase (*MT-ATP6*, *ATP5A1*, *ATPAF2* and *TMEM70*), inner membrane proteostasis (*YME1L*) and inner membrane dynamics (*OPA1* and *ATAD3A*), all of which are associated with PMDs with a wide range of phenotypes. Although these disorders typically involve eye, brain, muscle and heart symptoms, the critical pathological link to inflammation and immune signalling is that patient phenotypes often show subacute worsening upon infections; however, the involvement of cGAS-STING/type I IFN involvement in this observation has not been addressed.^52-55^

These findings have been translated into mitochondrial disease models. For example, *Opa1* knockout (KO) mice exhibit systemic inflammation triggered by mtDNA release and activation of TLR9, which leads directly to growth defects, although this appears to be untested in patients with *OPA1* variants.^16^ Patients harbouring pathogenic *ATAD3A* variants demonstrated increased type I interferon signalling in blood, with studies of patient-derived cells revealing that accumulation of cytosolic mtDNA drives cGAS/STING activation. Heterozygous *OPA1* mutations commonly cause autosomal dominant optic atrophy, whereas recessive *OPA1* mutations cause severe lethal infantile mitochondrial encephalomyopathy, hypertrophic cardiomyopathy and mtDNA depletion in muscle.^56^ Some dominant *OPA1* and *ATAD3A* variants are associated with more severe, systemic disorders, but whether mtDNA-mediated inflammatory signalling contributes to these more extensive phenotypes remains unknown. A recent screen identified that disruption of cardiolipin biosynthesis could rescue some of the phenotypes in cells from patients with dominant *OPA1* variants that cause systemic disease, highlighting the potential involvement of mtDNA-mediated inflammation in pathology.^17^ These data suggest that mtDNA release and inflammatory response might also contribute to other defects of mitochondrial membrane maintenance and cardiolipin metabolism (e.g. Barth syndrome and Senger’s syndrome), and future studies are needed to explore this possibility.

In addition to OPA1, wider mitochondrial fusion and fission machinery is involved in mtDNA maintenance, highlighting the importance of mitochondrial dynamics in regulating mtDNA release.^57,58^ OMM fusion is mediated by the GTPases mitofusin 1 and 2 (MFN1/2). Heterozygous missense pathogenic variants in *MFN2* lead to the development of the axonal peripheral neuropathy Charcot–Marie–Tooth 2A (CMT2A), causing progressive muscle weakness and sensory loss. A novel *MFN2* variant (p.Q367H) has been identified in a patient with distal myopathy, and it was shown to induce mtDNA release and activation of TLR9 and cGAS–STING pathways.^59^ Conditional *Mfn2*-KO in adult mice dopaminergic neurons causes mitochondrial fragmentation, disrupted cristae structure, inflammation through upregulation of genes activated by NF-κB and cGAS–STING, and activation of microglia.^19^ In mouse skeletal muscle, loss of mitochondrial fusion (Mfn1/2) and fission (Fis1 and Drp1) proteins activates NF-κB and type I interferon signalling, leading to muscle atrophy that is prevented by anti-inflammatory treatment.^18,20^ It was recently demonstrated that Drp1 depletion induces an endosome-mediated mtDNA disposal pathway that, when overwhelmed, promotes mtDNA release into the cytosol.^24^

## Mitochondrial DNA transcription and replication machinery

Mitochondria contain multiple copies of circular mtDNA compacted in nucleoprotein structures called nucleoids, which regulate mtDNA localization, distribution and expression. mtDNA replication is regulated autonomously by nuclear-encoded enzymes, facilitating initiation, elongation and termination.^60,61^ In addition, nucleoids have an important role in mtDNA maintenance, quantity and integrity and are linked to mitochondrial health.^62,63^ The major structural constituent of mtDNA nucleoids is the mitochondrial transcription factor A (TFAM), a nuclear-encoded protein that enables nucleoid compaction, regulating its architecture, mtDNA replication and transcription.^64^ Depletion of *Tfam* in mice is associated with enlarged nucleoids,^23,24^ mtDNA release and trafficking through endosomal pathways,^8^ and inflammation through cGAS–STING.^8,23,24^ Furthermore, in primed lipopolysaccharide-stimulated macrophages, oxidized TFAM facilitates the synthesis of new mtDNA, which triggers NLRP3 inflammasome complex activation.^22^ Targeted loss of *Tfam* from tubules caused kidney fibrosis and, strikingly, concomitant *Sting* knockout rescued inflammatory and fibrotic phenotypes.^23^ There are only two reported patients with pathogenic biallelic *TFAM* mutations, who both presented with neonatal liver failure (cirrhosis, steatosis and cholestasis) and mtDNA depletion with enlarged nucleoids.^65^ The fast progression of liver fibrosis has been suggested to be related to mtDNA release and inflammation.^66^ Additional mutations affecting *TFAM* expression revealed a loss of mtDNA stability and a type 1 IFN response.^30^ Circulating mtDNA and mitochondria-derived damage-associated molecular patterns are markedly increased in patients with non-alcoholic steatohepatitis and significant liver fibrosis, further strengthening the molecular link between liver involvement and mtDNA release-related inflammation; however, the causal relationship between these events needs further clarification.^66^

Mutations in mitochondrial DNA polymerase gamma (*POLG*), which facilitates mtDNA replication and repair, are among the most frequent causes of PMDs and are associated with a broad range of clinical presentations primarily affecting the brain, skeletal muscle and liver. This ranges from severe paediatric epileptic encephalopathy with liver failure (Alpers syndrome) to adult-onset neuropathy, ataxia and ophthalmoparesis or late-onset chronic progressive external ophthalmoplegia.^67^ Fibroblasts of patients with a common *POLG* mutation (W748S) causing mitochondrial recessive ataxia syndrome exhibit decreased mtDNA release and a dampened early immune response to viral infection. This compromises the activation of IFN antiviral response, but exacerbates later inflammatory responses, which might contribute to the worsening clinical manifestations during infection.^68^ Consistent with these findings, mice carrying the equivalent mutation to the human mitochondrial recessive ataxia syndrome mutation develop severe brain and liver disease upon viral infection, with exacerbated inflammation and loss of GABAergic neurons,^68^ suggesting that the release of mtDNA is crucial in the fight against viral infection. Further linking POLG with immunity, the mutator mouse carries a pathogenic exonuclease domain *Polg* variant, which causes mtDNA point mutations and deletions,^69^ exhibits exacerbated type I IFN and inflammatory responses mediated by the cGAS–STING pathway when treated with lipopolysaccharide, which mimics Gram-negative bacterial infection.^31^ Based on these data, the involvement of mtDNA release in *POLG*-related disease requires additional investigation.

To terminate mtDNA replication, mitochondrial topoisomerase 1 (TOP1MT) is involved in relieving mtDNA supercoiling.^61^ A pathogenic *TOP1MT* mutation caused autoimmune diseases, such as systemic lupus erythematosus and rheumatoid arthritis, with increased cytosolic mtDNA and type I IFN expression in several members of a consanguineous family, suggesting a possible contribution of mtDNA release in the development of these patients.^45^

## Nucleotide supply for mitochondrial DNA

The mitochondrial DNA maintenance machinery also encompasses enzymes facilitating the transport and biosynthesis of nucleotide pools.^70,71^ Thymidine kinase 2 (TK2) phosphorylates pyrimidine nucleosides, deoxycytidine and deoxythymidine, and *TK2* mutations cause severe early-onset or progressive late-onset mitochondrial myopathy with mtDNA depletion.^72^ An inflammatory response has been reported in skeletal muscle biopsies of patients with *TK2* mutations, showing infiltration of macrophages and overexpression of inflammatory and interferon-regulated genes.^27,73^ However, the underlying triggers of this immune response are still unexplored.

## Mitochondrial DNA quality control and degradation

The integrity of mtDNA is maintained through finely tuned and coordinated replication, repair and degradation. Nucleases play essential roles in these processes by regulating mitochondrial gene expression through the control of mtDNA and mtRNA levels and by participating in the repair of damaged mtDNA.^74^ Cellular RNases are key enzymes regulating mRNA processing and gene expression through interaction with the transcriptome. Mutations in nuclear-encoded mitochondrial RNAse enzymes, RNASEH2A and RNASEH2B, lead to Aicardi–Goutières syndrome, a rare condition characterized by neurological and immunological features linked to released mtDNA. Cell lines with these defects show changes in mitochondrial cristae organization, and increased level of reactive oxygen species and accelerated VDAC oligomer assembly in the outer mitochondrial membrane. This triggers mtDNA release, causing inflammation through both the cGAS–STING and the TLR9–NF-κB pathways.^28^

Another crucial component of the mitochondrial nuclease family is the mitochondrial genome maintenance exonuclease 1 (MGME1), which cleaves single-stranded DNA substrates and DNA flaps.^75^ Its absence causes an increase of single-stranded intermediates and short mtDNA fragments owing to halted replication, suggesting a role for the enzyme in the termination of mitochondrial replication.^29,75^ Biallelic mutations in *MGME1* result in severe multisystem neurological disease, with chronic progressive external ophthalmoplegia, myopathy, gastrointestinal and respiratory dysfunction.^75^Instability of mtDNA was confirmed in mice lacking *Mgme1*, showing a basal increase in single-stranded DNA. These animals develop chronic progressive nephropathy, with immune infiltrates and high levels of circulating inflammatory cytokines.^29^ Recent work further demonstrated that loss of MGME1 promotes aberrant ribonucleotide incorporation into mtDNA, leading to its cytosolic release, activation of the cGAS–STING pathway, and age-dependent inflammatory responses that drive renal failure in this mouse model.^76^

Mitochondria and mtDNA are removed through lysosomal degradation, in a specialized form of autophagy called mitophagy. Impaired mitophagy has been linked with cytosolic mtDNA release in several models of autoimmunity and ageing, suggesting the importance of regulation of this pathway in inflammation.^41,42,44^ In one mitophagy pathway, typically triggered by extensive mitochondrial damage, the mitochondrial ubiquitin kinase PINK1 and the E3 ubiquitin ligase Parkin drive the labelling process to eliminate dysfunctional mitochondria. Mutations in these genes cause early-onset Parkinson’s disease, where inflammation has been recognized as a crucial component of the pathophysiology.^77^ Interestingly, Pink/Parkin negatively regulates the formation of mitochondria-derived vesicles and the targeting of mitochondrial components to the multivesicular bodies .^17^ The link between mtDNA release, inflammation and Parkinson’s disease has been widely studied elsewhere.^43,78^

Given that mtDNA can be trafficked to lysosomes, this organelle is an important regulator of mtDNA release. DNase2, a nuclease that takes part in the autophagic process, cleaves DNA substrates in the lysosome, preventing an inflammatory response upon apoptosis or stress-induced degradation of mtDNA.^79^ Patients carrying biallelic *DNASE2* loss-of-function mutations present with an early-onset anaemia, recurrent fever, proteinuria and kidney disease, accompanied by DNA accumulation in lysosomes and a type I IFN autoimmune response.^80,81^ This evidences the overlap between mitochondrial disease and inborn errors of immunity and suggests that unbalanced mtDNA biogenesis and degradation are intimately linked to the activation of the innate immune inflammatory response, which is activated when degradation mechanisms, such as autophagy/mitophagy, fail. In support of this, mtDNA escapes autophagy in mice depleted in *Dnase2*, leading to cytosolic accumulation of mtDNA, and immune infiltration characterized by cytokine secretion and TLR9–NF-κB signalling. Furthermore, the model showed organ failure attributable to hepato- and cardiotoxic effects of the inflammation.^25,26^

## Mitochondrial proteostasis and mtDNA release

Conservation of a healthy mitochondrial proteome relies on correct protein import through both membranes, protein folding and quality control made by chaperones and proteases, and a correct balance in mitochondrial transcription–translation coupling.^82^ Failure of the mitochondrial proteostasis machinery has pleiotropic effects, including the activation of innate immune pathways.^83,84^ An example of this is the above-mentioned mitochondrial protease YME1L that has been implicated in a recent CRISPR screen, mapping mitochondrial components whose depletion induced cGAS/STING-dependent type I IFN signalling via pyrimidine-dependent mtDNA release.^15,54^ Another important protease in the mitochondrial matrix is LonP1, which degrades unfolded and damaged proteins. Homozygous recessive variants of *LONP1* have been related to the development of cerebral, ocular, dental, auricular and skeletal syndrome.^85^ Although this phenotype diverges from typical mitochondrial disease symptoms, cases with more usual mitochondrial phenotypes, including myopathy, OXPHOS dysfunction and mtDNA depletion, have been reported.^86,87^ LONP1 has been proposed to degrade TFAM, and *LONP1* mutations dysregulate TFAM and mtDNA levels, leading to the classical PMD phenotype.^87,88^ Interestingly, high levels of LonP1 have been found in CD4^+^ T cells obtained from a mouse model of systemic lupus erythematosus, where increased cytosolic mtDNA and activation of cGAS–STING–TBK1 inflammatory pathways mediate the immune activation.^89^ However, how LonP1 determines the mtDNA release has not been explored.

The caseinolytic mitochondrial matrix peptidase proteolytic subunit (ClpP) is part of the ClpXP complex, a mitochondrial proteasome-like cylinder that degrades misfolded proteins in the mitochondrial matrix. Autosomal recessive mutations in *CLPP* have been identified in patients with ovarian failure and sensorineural hearing loss (Perrault syndrome), but some patients also present with ataxia, learning disability and peripheral neuropathy, with increased mtDNA observed in patient fibroblasts.^90,91^ The *ClpP* knockout mouse model resembles human phenotypes with increased mtDNA levels, cGAS–STING activation and a type I IFN response.^21,92^ Other causes of Perrault syndrome are various defects of mitochondrial protein synthesis^93,94^ or, rarely, mtDNA replication.^95^

How mtDNA reaches the cytosol under proteolytic stress is not fully understood; however, an increase in membrane permeabilization seems to be one of the driving mechanisms. The mPTP, a putative channel that mediates the increase in permeability of mitochondrial membranes to ions and other molecules, has been suggested to mediate the mtDNA release and activation of cytosolic DNA sensors under mitochondrial stressors.^96-98^ The mitochondrial inner membrane m-AAA protease SPG7, an IMM metalloprotease and its interacting partner, AFG3L2, have been identified as regulators of the mPTP, and mutations in both genes can cause spastic ataxia with or without optic neuropathy. Dysregulation of this complex promotes mtDNA leakage into the cytosol and triggers an inflammatory response.^38^ Although impairment in mPTP opening has been observed in patient-derived cells, the impact of pathogenic variants in *SPG7* and *AFG3L2* on innate immune signalling remains unexplored.

## Mitochondrial translation

Mitochondrial translation is a well-controlled process requiring nuclear-encoded factors for its initiation, elongation and termination, and specific factors are needed for processing, modifying and amino-acylating mitochondrial tRNAs and for forming the mitochondrial ribosome.^99^ Mutations in the mtDNA-encoded tRNA^Leu^ (*MT-TL1*) and tRNA^Lys^ (*MT-TK*) are associated with mitochondrial encephalomyopathy, lactic acidosis, and stroke-like episodes (MELAS) and myoclonic epilepsy with ragged fibres (MERRF) syndromes, respectively, which are among the most common causes of mitochondrial diseases.

It has been shown that, in addition to the signalling role of mtDNA within the cell, it can also be released outside the cell. Circulating cell-free mtDNA is often increased in these conditions, particularly in MELAS, where baseline levels are high, and there is a further increase following a stroke-like episode.^9,100^ Circulating cell-free mtDNA showed a correlation with circulating inflammatory cytokines,^101^ and RNA-sequencing analysis revealed activation of type I IFN and antiviral response in MELAS patients and other mitochondrial disease models.^102^ Furthermore, defects in mtDNA distribution and an increase in DNA-sensing pathway proteins, such as IRF3, have been reported in skeletal muscle biopsies of a cohort of MELAS and chronic progressive external ophthalmoplegia patients,^32^ implying a role of these pathways in the pathomechanism. However, inflammatory responses and mtDNA release have not been reported in other defects of mitochondrial protein synthesis. Whether this reflects a distinct underlying mechanism with a less prominent role for mtDNA release or simply the lack of systematic studies across a broader range of mitochondrial translation defects remains to be determined.

## Respiratory chain subunit and assembly genes

Mitochondrial respiratory chain subunits and their nuclear-encoded assembly genes are well characterized and form a majority of the mitochondrial proteome.^103,104^ We found evidence in two of these gene defects (*NDUFS4* and *COX10*); however, for the majority of these conditions, it is unknown whether inflammation triggered by mtDNA contributes to mitochondrial dysfunction. Most data on the role of mtDNA-related immune response have become available for mutations of the complex I subunit gene *NDUFS4*, where neuroinflammation is thought to worsen neurodegeneration.^35^

Studies on *Ndufs4*-KO mice identified IFN-γ at disease onset, which increased in parallel with the progression of Leigh syndrome, suggesting that IFN-γ-targeting therapies might provide some benefits.^11^ However, IFN-γ is a type II interferon, and its association with mtDNA release has been reported only rarely. Chronic neuroinflammation triggered by interleukin-6 worsened the disease in female but not in male *Ndufs4*-KO mice, associated with an abnormal microglial response.^105^ Peripheral macrophages in brainstem lesions of these mice suggest their causal role in the CNS lesions.^36^ In a mouse model of the complex IV assembly cofactor Cox10, kidney cells show enhanced expression of interferon-stimulated genes and antiviral signalling pathways through cGAS–STING, which has been linked to autoimmune nephrotoxicity in mutant mice.^33^

Although not directly encoding for the respiratory chain, frataxin is a protein involved in iron metabolism and assembles iron–sulphur clusters essential for the electron transport chain. Mutations in frataxin are responsible for Friedreich’s ataxia, the most common hereditary ataxia, and induce mtDNA release and a type I interferon response via cGAS–STING sensing.^34^

There is limited information available on mtDNA release and coupled sterile inflammation in diseases of other mitochondrial translation factors, OXPHOS subunits, carriers, biosynthetic enzymes and cofactors, although these are common causes of PMD.^106^ Future research will determine whether mtDNA release and inflammation are truly less relevant in these diseases or whether they have simply not yet been investigated and might, in fact, contribute to their tissue-specific presentations.

## Conclusions

The role of released mtDNA as a signalling molecule has been studied extensively in ageing, cancer and infectious and metabolic diseases. Evidence showing mtDNA release from the mitochondria and activation of innate immune signalling pathways is growing, both in mitochondrial disease models and in patient samples.^102^ The next stage is to translate this into an understanding of its contribution to pathology. This is exciting because it opens new ways to develop treatments that can inhibit downstream inflammatory pathways, which have been used successfully in other disorders where innate immunity has been shown to drive pathology.

**Figure 1:**
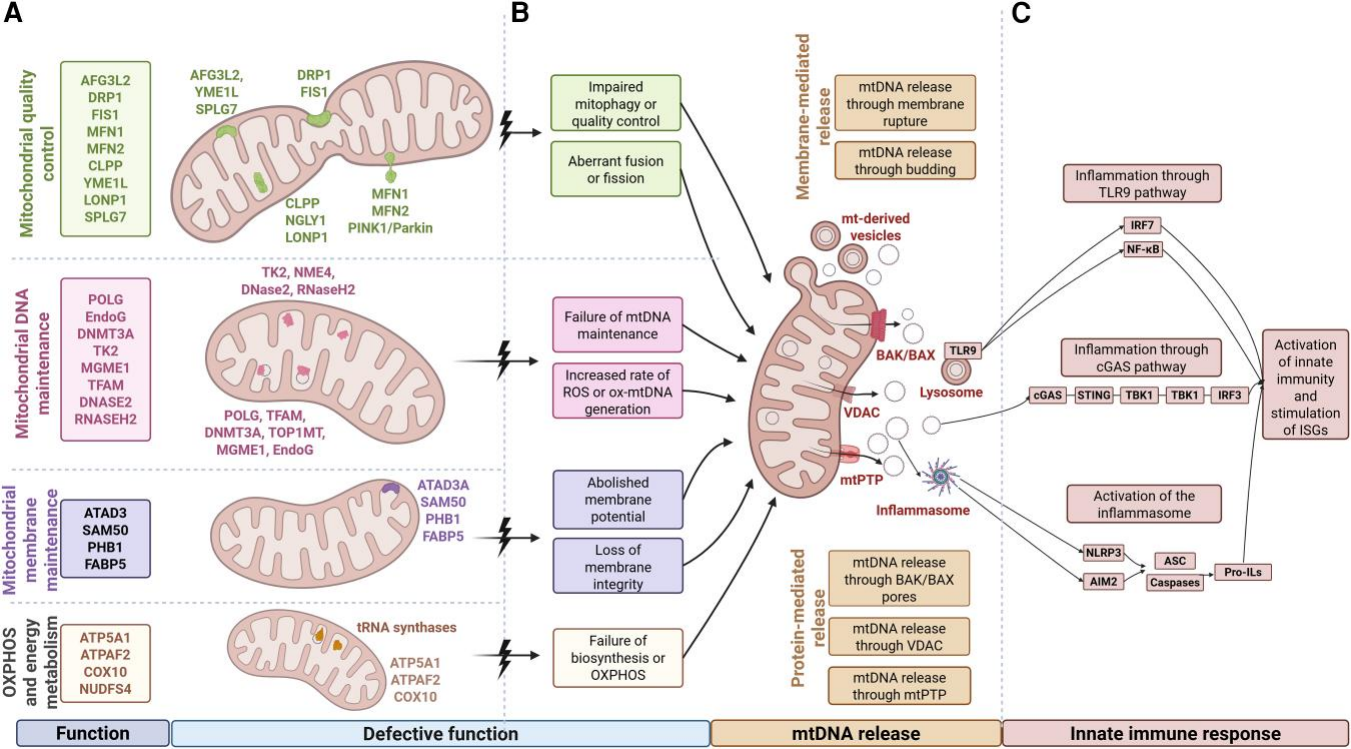
**Mechanisms linking mitochondrial dysfunction to mtDNA release and innate immune activation.** (**A**) Mitochondrial functional modules and associated factors. Schematic representation of key mitochondrial pathways whose perturbation predisposes to mtDNA instability and release. Mitochondrial quality control, mtDNA maintenance, mitochondrial membrane maintenance and OXPHOS and energy metabolism. Defects in these pathways undermine mitochondrial integrity and function. (**B**) Pathways leading from mitochondrial dysfunction to mtDNA release. Impaired mitochondrial quality control, aberrant fusion/fission dynamics, failure of mtDNA maintenance, excess reactive oxygen species or oxidized mtDNA production, loss of membrane potential or deficits in OXPHOS compromise mitochondrial homeostasis. These defects can result in mtDNA release into the cytosol through two broad mechanisms: membrane-mediated release, involving physical rupture of mitochondrial membranes or budding of mtDNA-containing vesicles; and protein-mediated release, including permeabilization by BAK/BAX macropores, VDAC oligomerization or opening of the mtPTP. (**C**) Innate immune pathways are activated by released mtDNA. Released mtDNA can be trafficked to lysosomes and/or activate different inflammatory pathways, such as TLR9 and NF-κB signalling, promoting pro-inflammatory cytokine production. Cytosolic mtDNA also activates the cGAS–STING pathway, inducing TBK1- and IRF3-dependent interferon signalling and expression of interferon-stimulated genes (ISGs). In parallel, oxidized or mislocalized mtDNA engages inflammasome sensors, such as NLRP3 and AIM2, leading to ASC and caspase activation and maturation of pro-interleukins. Together, these pathways link mitochondrial dysfunction to inflammation and broad innate immune activation. OXPHOS = oxidative phosphorylation; mtPTP = mitochondrial permeability transition pore. Created in BioRender. Szabo, M. (2026) https://BioRender.com/4ma0fmn.

Experimental models revealed three prominent ways for mtDNA release (mtPTP, BAK/BAX pores and VDAC) and highlighted that it can lead to inflammation, also via three pathways (cGAS–STING, TLR9–NF-κB and NLRP3–MAVS) (Fig. 1). Most studied mitochondrial models showed an activation of the type I interferon response,^8,26^ rendering it the most general pipeline for transmitting organellar stress. Evidence has been collected in diseases involving the regulation of mitochondrial cristae, biosynthesis of the inner and outer mitochondrial membranes, mtDNA maintenance genes and components of mitochondrial dynamics, which are more closely linked to the release of mtDNA. Furthermore, less explored mechanisms of mtDNA release and trafficking have been incorporated into the field, including mitochondrial and lysosomal Gasdermin pores and mtDNA trafficked in extracellular vesicles to neighbouring or distant cells.^107,108^

Despite this progress, several fundamental questions remain unanswered, and it is still unclear whether mtDNA release is simply a pathological event or represents an adaptive mechanism, helping cells to respond to external challenges, such as infections, immune triggers or metabolic stress, to preserve homeostasis. Answering these questions will be essential for bridging the gap between mitochondrial dysfunction and clinical phenotypes. Such advances will deepen our mechanistic understanding but also provide platforms for biomarker discovery and treatment development in PMDs and, more broadly, in common metabolic and neurological disorders associated with mitochondrial dysfunction.

## Supplementary Material

awag037_Supplementary_Data

## References

[1] Gorman GS, Chinnery PF, DiMauro S, et al Mitochondrial diseases. Nat Rev Dis Primers. 2016;2:16080. 27775730 10.1038/nrdp.2016.80

[2] Burr SP, Chinnery PF. Origins of tissue and cell-type specificity in mitochondrial DNA (mtDNA) disease. Hum Mol Genet. 2024;33:R3–R11. 38779777 10.1093/hmg/ddae059 PMC11112380

[3] Neeve VCM, Samuels DC, Bindoff LA, et al What is influencing the phenotype of the common homozygous polymerase-γ mutation p.Ala467Thr? Brain. 2012;135:3614–3626. 23250882 10.1093/brain/aws298 PMC3525059

[4] Marchi S, Guilbaud E, Tait SWG, Yamazaki T, Galluzzi L. Mitochondrial control of inflammation. Nat Rev Immunol. 2023;23:159–173. 35879417 10.1038/s41577-022-00760-x PMC9310369

[5] VanPortfliet JJ, Chute C, Lei Y, Shutt TE, West AP. Mitochondrial DNA release and sensing in innate immune responses. Hum Mol Genet. 2024;33:R80–R91. 38779772 10.1093/hmg/ddae031 PMC11112387

[6] West AP, McGuire PJ. Tipping the balance: Innate and adaptive immunity in mitochondrial disease. Curr Opin Immunol. 2025;95:102566. 40424975 10.1016/j.coi.2025.102566 PMC12210220

[7] Rocha M, Apostolova N, Diaz-Rua R, Muntane J, Victor VM. Mitochondria and T2D: Role of autophagy, ER stress, and inflammasome. Trends Endocrinol Metab. 2020;31:725–741. 32265079 10.1016/j.tem.2020.03.004

[8] West AP, Khoury-Hanold W, Staron M, et al Mitochondrial DNA stress primes the antiviral innate immune response. Nature. 2015;520:553–557. 25642965 10.1038/nature14156 PMC4409480

[9] Maresca A, Del Dotto V, Romagnoli M, et al Expanding and validating the biomarkers for mitochondrial diseases. J Mol Med (Berl). 2020;98:1467–1478. 32851462 10.1007/s00109-020-01967-y PMC7524861

[10] Trumpff C, Michelson J, Lagranha CJ, et al Stress and circulating cell-free mitochondrial DNA: A systematic review of human studies, physiological considerations, and technical recommendations. Mitochondrion. 2021;59:225–245. 33839318 10.1016/j.mito.2021.04.002 PMC8418815

[11] Hanaford AR, Khanna A, James K, et al Interferon-gamma contributes to disease progression in the *Ndufs4*(−/−) model of Leigh syndrome. Neuropathol Appl Neurobiol. 2024;50:e12977. 38680020 10.1111/nan.12977 PMC12952563

[12] Hanaford AR, Khanna A, Truong V, et al Disruption of adaptive immunity does not attenuate disease in the *Ndufs4*(-/-) model of Leigh syndrome. PLoS One. 2025;20:e0324268. 40493653 10.1371/journal.pone.0324268 PMC12151442

[13] Lehmann J, Giaglis S, Kyburz D, Daoudlarian D, Walker UA. Plasma mtDNA as a possible contributor to and biomarker of inflammation in rheumatoid arthritis. Arthritis Res Ther. 2024;26:97. 38715082 10.1186/s13075-024-03329-2 PMC11075188

[14] Lepelley A, Della Mina E, Van Nieuwenhove E, et al Enhanced cGAS-STING-dependent interferon signaling associated with mutations in ATAD3A. J Exp Med. 2021;218:e20201560. 34387651 10.1084/jem.20201560 PMC8374862

[15] Sprenger HG, MacVicar T, Bahat A, et al Cellular pyrimidine imbalance triggers mitochondrial DNA-dependent innate immunity. Nat Metab. 2021;3:636–650. 33903774 10.1038/s42255-021-00385-9 PMC8144018

[16] Rodríguez-Nuevo A, Díaz-Ramos A, Noguera E, et al Mitochondrial DNA and TLR9 drive muscle inflammation upon Opa1 deficiency. EMBO J. 2018;37:e96553. 29632021 10.15252/embj.201796553 PMC5978453

[17] Todkar K, Chikhi L, Desjardins V, El-Mortada F, Pépin G, Germain M. Selective packaging of mitochondrial proteins into extracellular vesicles prevents the release of mitochondrial DAMPs. Nat Commun. 2021;12:1971. 33785738 10.1038/s41467-021-21984-w PMC8009912

[18] Irazoki A, Gordaliza-Alaguero I, Frank E, et al Disruption of mitochondrial dynamics triggers muscle inflammation through interorganellar contacts and mitochondrial DNA mislocation. Nat Commun. 2023;14:108. 36609505 10.1038/s41467-022-35732-1 PMC9822926

[19] Filograna R, Lee S, Tiklova K, et al Mitochondrial dysfunction in adult midbrain dopamine neurons triggers an early immune response. PLoS Genet. 2021;17:e1009822. 34570766 10.1371/journal.pgen.1009822 PMC8496783

[20] Park S, Won JH, Hwang I, Hong S, Lee HK, Yu JW. Defective mitochondrial fission augments NLRP3 inflammasome activation. Sci Rep. 2015;5:15489. 26489382 10.1038/srep15489 PMC4614538

[21] Torres-Odio S, Lei Y, Gispert S, et al Loss of mitochondrial protease CLPP activates type I IFN responses through the mitochondrial DNA–cGAS–STING signaling axis. J Immunol. 2021;206:1890–1900. 33731338 10.4049/jimmunol.2001016 PMC8026707

[22] Zhong Z, Liang S, Sanchez-Lopez E, et al New mitochondrial DNA synthesis enables NLRP3 inflammasome activation. Nature. 2018;560:198–203. 30046112 10.1038/s41586-018-0372-z PMC6329306

[23] Chung KW, Dhillon P, Huang S, et al Mitochondrial damage and activation of the STING pathway lead to renal inflammation and fibrosis. Cell Metab. 2019;30:784–799.e5. 31474566 10.1016/j.cmet.2019.08.003 PMC7054893

[24] Newman LE, Weiser Novak S, Rojas GR, et al Mitochondrial DNA replication stress triggers a pro-inflammatory endosomal pathway of nucleoid disposal. Nat Cell Biol. 2024;26:194–206. 38332353 10.1038/s41556-023-01343-1 PMC11026068

[25] Oka T, Hikoso S, Yamaguchi O, et al Mitochondrial DNA that escapes from autophagy causes inflammation and heart failure. Nature. 2012;485:251–255. 22535248 10.1038/nature10992 PMC3378041

[26] Saito Y, Hikita H, Nozaki Y, et al DNase II activated by the mitochondrial apoptotic pathway regulates RIP1-dependent non-apoptotic hepatocyte death via the TLR9/IFN-β signaling pathway. Cell Death Differ. 2019;26:470–486. 29855540 10.1038/s41418-018-0131-6 PMC6370801

[27] Jou C, Nascimento A, Codina A, et al Pathological features in paediatric patients with TK2 deficiency. Int J Mol Sci. 2022;23:11002. 36232299 10.3390/ijms231911002 PMC9570075

[28] Dragoni F, Garau J, Sproviero D, et al Characterization of mitochondrial alterations in Aicardi–Goutières patients mutated in *RNASEH2A* and *RNASEH2B* genes. Int J Mol Sci. 2022;23:14482. 36430958 10.3390/ijms232214482 PMC9692803

[29] Milenkovic D, Sanz-Moreno A, Calzada-Wack J, et al Mice lacking the mitochondrial exonuclease MGME1 develop inflammatory kidney disease with glomerular dysfunction. PLoS Genet. 2022;18:e1010190. 35533204 10.1371/journal.pgen.1010190 PMC9119528

[30] Cobo I, Tanaka TN, Mangalhara KC, et al DNA methyltransferase 3 alpha and TET methylcytosine dioxygenase 2 restrain mitochondrial DNA-mediated interferon signaling in macrophages. Immunity. 2022;55:1386–1401.e10. 35931086 10.1016/j.immuni.2022.06.022 PMC9718507

[31] Lei Y, Guerra Martinez C, Torres-Odio S, et al Elevated type I interferon responses potentiate metabolic dysfunction, inflammation, and accelerated aging in mtDNA mutator mice. Sci Adv. 2021;7:eabe7548. 34039599 10.1126/sciadv.abe7548 PMC8153723

[32] Zhao X, Yu M, Zhao Y, et al Circulating cell-free mtDNA release is associated with the activation of cGAS-STING pathway and inflammation in mitochondrial diseases. J Neurol. 2022;269:4985–4996. 35486214 10.1007/s00415-022-11146-3

[33] Baek JH, Gomez IG, Wada Y, Roach A, Mahad D, Duffield JS. Deletion of the mitochondrial Complex-IV cofactor Heme A:farnesyltransferase causes focal segmental glomerulosclerosis and interferon response. Am J Pathol. 2018;188:2745–2762. 30268775 10.1016/j.ajpath.2018.08.018 PMC6334261

[34] Cotticelli MG, Xia S, Truitt R, et al Acute frataxin knockdown in induced pluripotent stem cell-derived cardiomyocytes activates a type I interferon response. Dis Model Mech. 2022;16:dmm049497. 36107856 10.1242/dmm.049497 PMC9637271

[35] Aguilar K, Comes G, Canal C, Quintana A, Sanz E, Hidalgo J. Microglial response promotes neurodegeneration in the *Ndufs4 KO* mouse model of Leigh syndrome. Glia. 2022;70:2032–2044. 35770802 10.1002/glia.24234 PMC9544686

[36] Hanaford AR, Khanna A, Truong V, et al Peripheral macrophages drive CNS disease in the *Ndufs4*(−/−) model of Leigh syndrome. Brain Pathol. 2023;33:e13192. 37552802 10.1111/bpa.13192 PMC10580015

[37] Chen L, Dong J, Liao S, et al Loss of Sam50 in hepatocytes induces cardiolipin-dependent mitochondrial membrane remodeling to trigger mtDNA release and liver injury. Hepatology. 2022;76:1389–1408. 35313046 10.1002/hep.32471

[38] Liu H, Fan H, He P, et al Prohibitin 1 regulates mtDNA release and downstream inflammatory responses. EMBO J. 2022;41:e111173. 36245295 10.15252/embj.2022111173 PMC9753472

[39] He B, Yu H, Liu S, et al Mitochondrial cristae architecture protects against mtDNA release and inflammation. Cell Rep. 2022;41:111774. 36476853 10.1016/j.celrep.2022.111774

[40] Field CS, Baixauli F, Kyle RL, et al Mitochondrial integrity regulated by lipid metabolism is a cell-intrinsic checkpoint for Treg suppressive function. Cell Metab. 2020;31:422–437.e5. 31883840 10.1016/j.cmet.2019.11.021 PMC7001036

[41] Rai P, Janardhan KS, Meacham J, et al IRGM1 links mitochondrial quality control to autoimmunity. Nat Immunol. 2021;22:312–321. 33510463 10.1038/s41590-020-00859-0 PMC7906953

[42] Irazoki A, Martinez-Vicente M, Aparicio P, et al Coordination of mitochondrial and lysosomal homeostasis mitigates inflammation and muscle atrophy during aging. Aging Cell. 2022;21:e13583. 35263007 10.1111/acel.13583 PMC9009131

[43] Bueno M, Zank D, Buendia-Roldán I, et al PINK1 attenuates mtDNA release in alveolar epithelial cells and TLR9 mediated profibrotic responses. PLoS One. 2019;14:e0218003. 31170232 10.1371/journal.pone.0218003 PMC6553779

[44] Yang K, Huang R, Fujihira H, Suzuki T, Yan N. N-glycanase NGLY1 regulates mitochondrial homeostasis and inflammation through NRF1. J Exp Med. 2018;215:2600–2616. 30135079 10.1084/jem.20180783 PMC6170171

[45] Al Khatib I, Deng J, Lei Y, et al Activation of the cGAS-STING innate immune response in cells with deficient mitochondrial topoisomerase TOP1MT. Hum Mol Genet. 2023;32:2422–2440. 37129502 10.1093/hmg/ddad062 PMC10360396

[46] Xian H, Watari K, Sanchez-Lopez E, et al Oxidized DNA fragments exit mitochondria via mPTP- and VDAC-dependent channels to activate NLRP3 inflammasome and interferon signaling. Immunity. 2022;55:1370–1385.e8. 35835107 10.1016/j.immuni.2022.06.007 PMC9378606

[47] Kim J, Gupta R, Blanco LP, et al VDAC oligomers form mitochondrial pores to release mtDNA fragments and promote lupus-like disease. Science. 2019;366:1531–1536. 31857488 10.1126/science.aav4011 PMC8325171

[48] Ježek P, Jabůrek M, Holendová B, Engstová H, Dlasková A. Mitochondrial cristae morphology reflecting metabolism, superoxide formation, redox homeostasis, and pathology. Antioxid Redox Signal. 2023;39:635–683. 36793196 10.1089/ars.2022.0173 PMC10615093

[49] Anand R, Reichert AS, Kondadi AK. Emerging roles of the MICOS complex in cristae dynamics and biogenesis. Biology (Basel). 2021;10:600. 34209580 10.3390/biology10070600 PMC8301002

[50] Sen A, Kallabis S, Gaedke F, et al Mitochondrial membrane proteins and VPS35 orchestrate selective removal of mtDNA. Nat Commun. 2022;13:6704. 36344526 10.1038/s41467-022-34205-9 PMC9640553

[51] McArthur K, Whitehead LW, Heddleston JM, et al BAK/BAX macropores facilitate mitochondrial herniation and mtDNA efflux during apoptosis. Science. 2018;359:eaao6047. 29472455 10.1126/science.aao6047

[52] Thompson K, Bianchi L, Rastelli F, et al Biallelic variants in *TAMM41* are associated with low muscle cardiolipin levels, leading to neonatal mitochondrial disease. HGG Adv. 2022;3:100097. 35321494 10.1016/j.xhgg.2022.100097 PMC8935507

[53] Tauchmannová K, Pecinová A, Houštěk J, Mráček T. Variability of clinical phenotypes caused by isolated defects of mitochondrial ATP synthase. Physiol Res. 2024;73(Suppl 1):S243–S278. 39016153 10.33549/physiolres.935407 PMC11412354

[54] Hartmann B, Wai T, Hu H, et al Homozygous *YME1L1* mutation causes mitochondriopathy with optic atrophy and mitochondrial network fragmentation. eLife. 2016;5:e16078. 27495975 10.7554/eLife.16078 PMC4991934

[55] Harel T, Yoon WH, Garone C, et al Recurrent de novo and biallelic variation of *ATAD3A*, encoding a mitochondrial membrane protein, results in distinct neurological syndromes. Am J Hum Genet. 2016;99:831–845. 27640307 10.1016/j.ajhg.2016.08.007 PMC5065660

[56] Spiegel R, Saada A, Flannery PJ, et al Fatal infantile mitochondrial encephalomyopathy, hypertrophic cardiomyopathy and optic atrophy associated with a homozygous *OPA1* mutation. J Med Genet. 2016;53:127–131. 26561570 10.1136/jmedgenet-2015-103361 PMC4752660

[57] Tábara LC, Burr SP, Frison M, et al MTFP1 controls mitochondrial fusion to regulate inner membrane quality control and maintain mtDNA levels. Cell. 2024;187:3619–3637.e27. 38851188 10.1016/j.cell.2024.05.017

[58] Ramos ES, Motori E, Brüser C, et al Mitochondrial fusion is required for regulation of mitochondrial DNA replication. PLoS Genet. 2019;15:e1008085. 31170154 10.1371/journal.pgen.1008085 PMC6553695

[59] Zaman M, Sharma G, Almutawa W, et al The MFN2 Q367H variant reveals a novel pathomechanism connected to mtDNA-mediated inflammation. Life Sci Alliance. 2025;8:e202402921. 40175090 10.26508/lsa.202402921 PMC11966011

[60] Falkenberg M . Mitochondrial DNA replication in mammalian cells: Overview of the pathway. Essays Biochem. 2018;62:287–296. 29880722 10.1042/EBC20170100 PMC6056714

[61] Basu U, Bostwick AM, Das K, Dittenhafer-Reed KE, Patel SS. Structure, mechanism, and regulation of mitochondrial DNA transcription initiation. J Biol Chem. 2020;295:18406–18425. 33127643 10.1074/jbc.REV120.011202 PMC7939475

[62] Lee SR, Han J. Mitochondrial nucleoid: Shield and switch of the mitochondrial genome. Oxid Med Cell Longev. 2017;2017:8060949. 28680532 10.1155/2017/8060949 PMC5478868

[63] Jenkins BC, Neikirk K, Katti P, et al Mitochondria in disease: Changes in shapes and dynamics. Trends Biochem Sci. 2024;49:346–360. 38402097 10.1016/j.tibs.2024.01.011 PMC10997448

[64] Kang I, Chu CT, Kaufman BA. The mitochondrial transcription factor TFAM in neurodegeneration: Emerging evidence and mechanisms. FEBS Lett. 2018;592:793–811. 29364506 10.1002/1873-3468.12989 PMC5851836

[65] Stiles AR, Simon MT, Stover A, et al Mutations in TFAM, encoding mitochondrial transcription factor A, cause neonatal liver failure associated with mtDNA depletion. Mol Genet Metab. 2016;119:91–99. 27448789 10.1016/j.ymgme.2016.07.001

[66] An P, Wei LL, Zhao S, et al Hepatocyte mitochondria-derived danger signals directly activate hepatic stellate cells and drive progression of liver fibrosis. Nat Commun. 2020;11:2362. 32398673 10.1038/s41467-020-16092-0 PMC7217909

[67] Rahman S, Copeland WC. *POLG*-related disorders and their neurological manifestations. Nat Rev Neurol. 2019;15:40–52. 30451971 10.1038/s41582-018-0101-0 PMC8796686

[68] Kang Y, Hepojoki J, Maldonado RS, et al Ancestral allele of DNA polymerase gamma modifies antiviral tolerance. Nature. 2024;628:844–853. 38570685 10.1038/s41586-024-07260-z PMC11041766

[69] Edgar D, Trifunovic A. The mtDNA mutator mouse: Dissecting mitochondrial involvement in aging. Aging (Albany NY). 2009;1:1028–1032. 20157586 10.18632/aging.100109 PMC2815752

[70] Shadel GS, Clayton DA. Mitochondrial DNA maintenance in vertebrates. Annu Rev Biochem. 1997;66:409–435. 9242913 10.1146/annurev.biochem.66.1.409

[71] El-Hattab AW, Craigen WJ, Scaglia F. Mitochondrial DNA maintenance defects. Biochim Biophys Acta Mol Basis Dis. 2017;1863:1539–1555. 28215579 10.1016/j.bbadis.2017.02.017

[72] Garone C, Taylor RW, Nascimento A, et al Retrospective natural history of thymidine kinase 2 deficiency. J Med Genet. 2018;55:515–521. 29602790 10.1136/jmedgenet-2017-105012 PMC6073909

[73] Kalko SG, Paco S, Jou C, et al Transcriptomic profiling of TK2 deficient human skeletal muscle suggests a role for the p53 signalling pathway and identifies growth and differentiation factor-15 as a potential novel biomarker for mitochondrial myopathies. BMC Genomics. 2014;15:91. 24484525 10.1186/1471-2164-15-91 PMC3937154

[74] Rong Z, Tu P, Xu P, et al The mitochondrial response to DNA damage. Front Cell Dev Biol. 2021;9:669379. 34055802 10.3389/fcell.2021.669379 PMC8149749

[75] Kornblum C, Nicholls TJ, Haack TB, et al Loss-of-function mutations in *MGME1* impair mtDNA replication and cause multisystemic mitochondrial disease. Nat Genet. 2013;45:214–219. 23313956 10.1038/ng.2501 PMC3678843

[76] Bahat A, Milenkovic D, Cors E, et al Ribonucleotide incorporation into mitochondrial DNA drives inflammation. Nature. 2025;647:726–734. 40993386 10.1038/s41586-025-09541-7 PMC12629987

[77] Dzamko N, Geczy CL, Halliday GM. Inflammation is genetically implicated in Parkinson’s disease. Neuroscience. 2015;302:89–102. 25450953 10.1016/j.neuroscience.2014.10.028

[78] Borsche M, König IR, Delcambre S, et al Mitochondrial damage-associated inflammation highlights biomarkers in *PRKN/PINK1* parkinsonism. Brain. 2020;143:3041–3051. 33029617 10.1093/brain/awaa246 PMC7586086

[79] Matsui H, Ito J, Matsui N, Uechi T, Onodera O, Kakita A. Cytosolic dsDNA of mitochondrial origin induces cytotoxicity and neurodegeneration in cellular and zebrafish models of Parkinson’s disease. Nat Commun. 2021;12:3101. 34035300 10.1038/s41467-021-23452-x PMC8149644

[80] Rodero MP, Tesser A, Bartok E, et al Type I interferon-mediated autoinflammation due to DNase II deficiency. Nat Commun. 2017;8:2176. 29259162 10.1038/s41467-017-01932-3 PMC5736616

[81] Hong Y, Capitani M, Murphy C, et al Janus kinase inhibition for autoinflammation in patients with *DNASE2* deficiency. J Allergy Clin Immunol. 2020;145:701–705.e8. 31775019 10.1016/j.jaci.2019.11.020

[82] Moehle EA, Shen K, Dillin A. Mitochondrial proteostasis in the context of cellular and organismal health and aging. J Biol Chem. 2019;294:5396–5407. 29622680 10.1074/jbc.TM117.000893 PMC6462515

[83] Moehlman AT, Youle RJ. Mitochondrial quality control and restraining innate immunity. Annu Rev Cell Dev Biol. 2020;36:265–289. 33021820 10.1146/annurev-cellbio-021820-101354

[84] Ahola S, Langer T, MacVicar T. Mitochondrial proteolysis and metabolic control. Cold Spring Harb Perspect Biol. 2019;11:a033936. 30670467 10.1101/cshperspect.a033936 PMC6601461

[85] Inui T, Anzai M, Takezawa Y, et al A novel mutation in the proteolytic domain of *LONP1* causes atypical CODAS syndrome. J Hum Genet. 2017;62:653–655. 28148925 10.1038/jhg.2017.11

[86] Hannah-Shmouni F, MacNeil L, Brady L, Nilsson MI, Tarnopolsky M. Expanding the clinical spectrum of *LONP1*-related mitochondrial cytopathy. Front Neurol. 2019;10:981. 31636596 10.3389/fneur.2019.00981 PMC6787162

[87] Peter B, Waddington CL, Oláhová M, et al Defective mitochondrial protease LonP1 can cause classical mitochondrial disease. Hum Mol Genet. 2018;27:1743–1753. 29518248 10.1093/hmg/ddy080 PMC5932559

[88] Matsushima Y, Goto Y, Kaguni LS. Mitochondrial Lon protease regulates mitochondrial DNA copy number and transcription by selective degradation of mitochondrial transcription factor A (TFAM). Proc Natl Acad Sci U S A. 2010;107:18410–18415. 20930118 10.1073/pnas.1008924107 PMC2972957

[89] Huang X, Liu Y, Ling G, Cao X. Mitochondrial Lon protease promotes CD4^+^ T cell activation by activating the cGAS-STING-TBK1 axis in systemic lupus erythematosus. Int Immunopharmacol. 2023;123:110519. 37531828 10.1016/j.intimp.2023.110519

[90] Theunissen TEJ, Szklarczyk R, Gerards M, et al Specific MRI abnormalities reveal severe Perrault syndrome due to CLPP defects. Front Neurol. 2016;7:203. 27899912 10.3389/fneur.2016.00203 PMC5110515

[91] Jenkinson EM, Rehman AU, Walsh T, et al Perrault syndrome is caused by recessive mutations in *CLPP*, encoding a mitochondrial ATP-dependent chambered protease. Am J Hum Genet. 2013;92:605–613. 23541340 10.1016/j.ajhg.2013.02.013 PMC3617381

[92] Gispert S, Parganlija D, Klinkenberg M, et al Loss of mitochondrial peptidase *Clpp* leads to infertility, hearing loss plus growth retardation via accumulation of CLPX, mtDNA and inflammatory factors. Hum Mol Genet. 2013;22:4871–4887. 23851121 10.1093/hmg/ddt338 PMC7108587

[93] Key J, Gispert S, Koornneef L, et al CLPP depletion causes diplotene arrest; underlying testis mitochondrial dysfunction occurs with accumulation of Perrault proteins ERAL1, PEO1, and HARS2. Cells. 2023;12:52. 10.3390/cells12010052 PMC9818230 36611846

[94] Pierce SB, Chisholm KM, Lynch ED, et al Mutations in mitochondrial histidyl tRNA synthetase *HARS2* cause ovarian dysgenesis and sensorineural hearing loss of Perrault syndrome. Proc Natl Acad Sci U S A. 2011;108:6543–6548. 21464306 10.1073/pnas.1103471108 PMC3081023

[95] Li H, Cao C, Lv Y. *TWNK* gene pathogenic variant and Perrault syndrome. Gene. 2025;965:149667. 40669787 10.1016/j.gene.2025.149667

[96] Ouyang W, Wang S, Yan D, et al The cGAS-STING pathway-dependent sensing of mitochondrial DNA mediates ocular surface inflammation. Signal Transduct Target Ther. 2023;8:371. 37735446 10.1038/s41392-023-01624-z PMC10514335

[97] Patrushev M, Kasymov V, Patrusheva V, Ushakova T, Gogvadze V, Gaziev AI. Release of mitochondrial DNA fragments from brain mitochondria of irradiated mice. Mitochondrion. 2006;6:43–47. 16413832 10.1016/j.mito.2005.12.001

[98] Kim J, Kim HS, Chung JH. Molecular mechanisms of mitochondrial DNA release and activation of the cGAS-STING pathway. Exp Mol Med. 2023;55:510–519. 36964253 10.1038/s12276-023-00965-7 PMC10037406

[99] Boczonadi V, Ricci G, Horvath R. Mitochondrial DNA transcription and translation: Clinical syndromes. Essays Biochem. 2018;62:321–340. 29980628 10.1042/EBC20170103 PMC6056718

[100] Trifunov S, Paredes-Fuentes AJ, Badosa C, et al Circulating cell-free mitochondrial DNA in cerebrospinal fluid as a biomarker for mitochondrial diseases. Clin Chem. 2021;67:1113–1121. 34352085 10.1093/clinchem/hvab091

[101] Zhou M, Zhang H, Xu X, Chen H, Qi B. Association between circulating cell-free mitochondrial DNA and inflammation factors in noninfectious diseases: A systematic review. PLoS One. 2024;19:e0289338. 38241222 10.1371/journal.pone.0289338 PMC10798522

[102] Warren EB, Gordon-Lipkin EM, Cheung F, et al Inflammatory and interferon gene expression signatures in patients with mitochondrial disease. J Transl Med. 2023;21:331. 37208779 10.1186/s12967-023-04180-w PMC10199642

[103] Fernandez-Vizarra E, Zeviani M. Mitochondrial disorders of the OXPHOS system. FEBS Lett. 2021;595:1062–1106. 33159691 10.1002/1873-3468.13995

[104] Rébeillé F, Alban C, Bourguignon J, Ravanel S, Douce R. The role of plant mitochondria in the biosynthesis of coenzymes. Photosynth Res. 2007;92:149–162. 17464574 10.1007/s11120-007-9167-z

[105] Aguilar K, Canal C, Comes G, et al Interleukin-6-elicited chronic neuroinflammation may decrease survival but is not sufficient to drive disease progression in a mouse model of Leigh syndrome. J Inflamm (Lond). 2024;21:1. 38212783 10.1186/s12950-023-00369-4 PMC10782699

[106] Madsen HB, Pease LI, Scanlan RL, et al The DNA repair enzyme, aprataxin, plays a role in innate immune signaling. Front Aging Neurosci. 2023;15:1290681. 38161589 10.3389/fnagi.2023.1290681 PMC10754971

[107] Miao N, Wang Z, Wang Q, et al Oxidized mitochondrial DNA induces gasdermin D oligomerization in systemic lupus erythematosus. Nat Commun. 2023;14:872. 36797275 10.1038/s41467-023-36522-z PMC9935630

[108] Lou P, Zhou X, Zhang Y, et al Harnessing tissue-derived mitochondria-rich extracellular vesicles (Ti-mitoEVs) to boost mitochondrial biogenesis for regenerative medicine. Sci Adv. 2025;11:eadt1318. 40668934 10.1126/sciadv.adt1318 PMC12266123

