# Supplementary material for: Mitochondrial DNA release and inflammation in mitochondrial disease pathogenesis
Source: Brain. 2026 Feb 2;149(6):1874–84. doi: 10.1093/brain/awag037 (PMC13233045; doi:10.1093/brain/awag037)
Supplement: awag037_Supplementary_Data [file awag037_supplementary_data.pdf]

Supplementary Material

Mitochondrial DNA Release and Inflammation in Mitochondrial Disease Pathogenesis

Marton Szabo[1†], Daniel Lagos[1†#], Emily Cross[1], Jack Collier[1], Rita Horvath[1#]

Systematic Review Protocol

We aimed to identify any relevant papers describing altered function of mitochondria or mitochondria-related proteins, causing impaired mitochondrial physiology-coupled mtDNA release and subsequent activation of innate immunity. Our systematic review is disclosed according to guidelines posited by the Preferred Reporting Items for Systematic Reviews and Meta-Analyses (PRISMA) Statement [1].

Literature Search Strategy

In order to include all relevant articles we searched PubMed with a heading including: ("mtDNA" or "mitochondrial DNA") AND (immun* OR inflamm*) AND (release OR escape OR transport OR cell-free OR vesicles OR mutation OR knock*) AND (muscle OR myopathy OR neuron OR neuropathy OR disease OR encephalopathy). We restricted our search to articles before 2011, as the first case of mitochondrial DNA release in the context of immunity was reported in 2011 by Nakahira et al. (2011) [2]. The search engine identified 1,948 articles, as of 6 May 2024, which were transferred to the Rayyan QCRI platform for subsequent screening and classification.

Inclusion Criteria

- Methodology relied on primary human cells with mitochondria-related mutation or other cell types induced with analogous mutation
- Animal models and human patients of primary mitochondrial diseases or secondary mitochondrial dysfunctions
- Confirmed mutation affecting mitochondrial activity, encoded in mitochondrial genome, or encoded in nuclear genome with relevant mitochondrial function

- Mutation induced mitochondrial DNA release in the context of impaired mitochondrial function criteria fulfilled
- Implications of released mitochondrial DNA were described (including mitophagy function, nucleoid morphology, mitochondrial membrane potential, OXPHOS activity, cristae dynamics)
- Confirmed relation of mitochondrial DNA release and activated pathways of innate immunity induced sterile inflammation

Exclusion Criteria

- Studies published before 2011 i.e. before the first reported association of mitochondrial and innate inflammation by Nakahire et al. (2011)
- Studies that are not in English
- Studies that were retracted or do not have the full text available
- Studies not about primary mitochondrial diseases or secondary mitochondrial dysfunctions
    - Describing mtDNA release and coupled immune response in the context of pre-existing non-mitochondria related chronic medical conditions (neurodegenerative disorders, cancer, autoimmune diseases, cardiovascular diseases)
    - Describing mtDNA release and coupled immune response in the context of an acute medical condition (injury, infection, sepsis)
    - Induced mitochondrial DNA release through non-genetic means (exercise, aging, drug treatment, surgical intervention)
- Studies not including immunological implications of mtDNA release
    - Assessing mitochondrial function in the context of physiological implications, diagnostic methods or biomarker studies
    - Describing mitochondrial function and inflammatory response without establishing a functional and/or morphological link between the processes
- Studies that reported on non-original data including: duplicate studies, case reports or review articles

Supplementary Table. Summary of excluded papers based on wrong population or wrong study design, showing the most prominent groups where n>30 and n>5, respectively for the two groups.

Wrong population	Wrong study design
Autoimmune (n = 154)	Other transcription/translation mutations (n =32)
Infection (n = 116)	Other OXPHOS mutations (n = 25)
Cardiovascular (n = 111)	LHON (n = 19)
Cancer (n = 82)	MELAS (n = 14)
Neurodegenerative (n = 79)	Other quality control mutations (n = 11)
Hepatological (n = 51)	Leigh syndrome (n = 8)
Senescence (n = 38)	
Diabetes (n = 33)	

Supplementary Figure. Screening process.

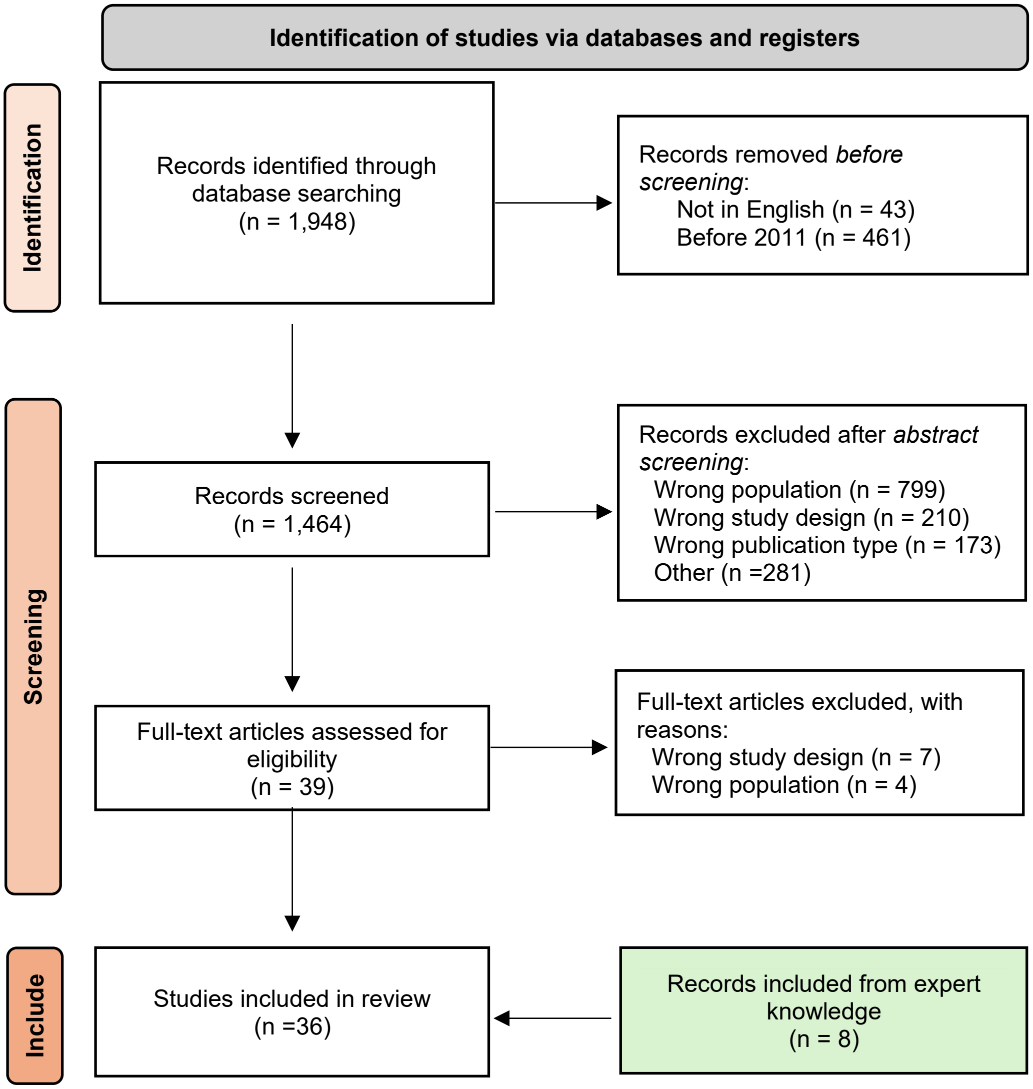

References

1. Moher D, Liberati A, Tetzlaff J, Altman DG, Group TP. Preferred Reporting Items for Systematic Reviews and Meta-Analyses: The PRISMA Statement. *PLOS Med*. 2009;6(7):e1000097. doi:10.1371/journal.pmed.1000097

2. Nakahira K, Haspel JA, Rathinam VAK, et al. Autophagy proteins regulate innate immune responses by inhibiting the release of mitochondrial DNA mediated by the NALP3 inflammasome. *Nat Immunol*. 2011;12(3):222-230. doi:10.1038/ni.1980
